# The Profile of T Helper Subsets in Bone Marrow Microenvironment Is Distinct for Different Stages of Acute Myeloid Leukemia Patients and Chemotherapy Partly Ameliorates These Variations

**DOI:** 10.1371/journal.pone.0131761

**Published:** 2015-07-02

**Authors:** Tian Tian, Shuang Yu, Lu Liu, Fuzhong Xue, Cunzhong Yuan, Min Wang, Chunyan Ji, Daoxin Ma

**Affiliations:** 1 Department of Hematology, Qilu Hospital, Shandong University, Jinan, China; 2 Department of Obstetrics and Gynecology, Qilu Hospital, Shandong University, Jinan, China; 3 Department of Epidemiology and Biostatistics, School of Public health, Shandong University, Jinan, China; University of Nebraska Medical Center, UNITED STATES

## Abstract

**Background:**

T helper (Th) cells immune regulation is important for the pathogenesis of acute myeloid leukemia (AML). Recurrent Th abnormalities in AML peripheral blood were reported, while the comprehensive status of various Th subsets is rarely investigated in bone marrow (BM) microenvironment which is the origin of AML leukemic blast cells.

**Methods:**

BM was extracted from 48 newly-diagnosed (ND), 34 complete-remission (CR), 19 relapsed-refractory AML patients and 15 controls. Slight iron deficiency anemia patients were used as controls. Th subsets frequencies were examined by flow cytometry. BM plasma Th-associated cytokines levels were determined by ELISA. The expression of key transcription factor was examined by RT-PCR.

**Results:**

Th22, Th17, Th1, Th2 cells, IL-22 and *RORC* expression were significantly decreased, while Treg cells, related cytokine IL-10 and transcription factor *Foxp3* were markedly elevated in ND compared to CR patients or controls. Meanwhile, the imbalanced Th1/Th2 and Th17/Treg ratio were observed in ND and relapsed-refractory patients. Negative correlation between Th1 or Th2 and peripheral WBC, between Th17/Treg or Th1/Th2 and leukemic blast existed in ND patients. Moreover, chemotherapy ameliorated these variations.

**Conclusion:**

Th subsets in BM are distinct for different stages of AML and chemotherapy partly ameliorates the abnormality. Our findings suggest that these cells and cytokines may be implicated in AML pathogenesis and provided therapeutic insights.

## Introduction

Acute myeloid leukemia (AML), characterized by the proliferation of clonal neoplastic myeloid precursor cells and impaired production of normal hematopoiesis, is one of the hematological malignancies with the highest incidence in adults [1–3]. Immune system disorder has been shown in the pathogenesis of AML. T cells immune, important for anti-tumor immunity, eliminates AML cells through releasing cytokines and cytotoxic substances. Parallelly, AML cells influence T cells differentiation and proliferation, and play an immunosuppressive role by releasing inhibitory cytokines or other kinds of mechanisms. T helper (Th) cells play a pivotal status in T cell immune system network. Previous researches on Th cells are limited to Th1 or Th2 subset. Th1 cells mediating cellular immunity develop in response to IL-12 and produce Interferon-γ (IFN-γ), while Th2 enhancing humoral immunity develop in response to IL-4 and produce IL-4, IL-5 and IL-13 [4,5]. Accumulating evidences indicate an imbalanced Th1/Th2 involved in the pathogenesis of solid tumors [6] as well as hematological malignancies [7,8]. However, researches on Th1/Th2 in bone marrow (BM) microenvironment are limited. Recently, studies have shown that Th cells also include Treg, Th17, Th22 and Th9 subsets.

Th17 and Treg are considered as another paired Th subsets, and Th17/Treg imbalance is found in many diseases. Tregs are characterized by constitutive expression of high-level CD25, and forkhead-winged helix transcription factor (*Foxp3*) is essential for Tregs development or function [9,10]. Tregs engage in maintaining natural self-tolerance and controlling immune responses to foreign antigens [11]. Several researches show the elevated levels of Tregs in several hematological malignancies including AML [8,12–14]. However, in AML BM microenvironment, the related researches are rare. Th17 cells, IL-17-producing CD4^+^ T cells, are considered as an early subset of effector CD4^+^ T cells produced directly from naïve CD4^+^ T cells [15,16]. Th17 cells and IL-17 have a regulatory role in normal hematopoiesis [17]. It has been established that Th17 cells participate in some autoimmune diseases and tumors [18–20]. Moreover, the previous results about circulating Th17 cells in AML are divergent [21], which promote us to do further researches to interpret its exact role.

Th22 are recently-identified CD4^+^ T cells that produce IL-22 but not IL-17 or IFN-γ. Th22 expresses chemokine (C-C motif) receptor 6 (CCR6), CCR4 and CCR10 but not CD161 [22]. Moreover, Th22 cells have low or undetectable expression of Th1 or Th17 transcription factor T-box expressed in T cells (*T-bet*) or Retinoic acid-related orphan receptor-C (*RORC*), while Aryl hydrocarbon receptor (*AHR*) has been considered to be the key transcription factor of Th22. In addition, naïve T cells differentiate towards Th22 phenotype in the presence of IL-6 and tumor necrosis factor-α (TNF-α) [22]. All of above provide strong evidence that Th22 represents an independent and terminally-differentiated Th subtype. The effector cytokine of Th22 is IL-22, which belongs to IL-10 cytokine family. Recent studies have implicated the involvement of IL-22 in the pathogenesis of autoimmune diseases, such as rheumatoid arthritis, Crohn’s disease, psoriasis and systemic lupus erythematosus [23] and some hematological diseases, such as myelodysplastic syndrome (MDS) and idiopathic thrombocytopenic purpura (ITP) [24,25]. However, till now, the specific roles of Th22 and IL-22 in the pathogenesis of AML are still unclear.

Recently, there has been renewed interest in IL-9-producing CD4^+^ T cells, including Th2, Th17 and Treg cells [26]. IL-9 production was first associated with Th2 phenotype and plays an important role in the pathogenesis of asthma and resistance to parasite [27]. Recently, studies showed the most consistent IL-9-producing T cells generated with the cytokines Transforming growth factor-β (TGF-β) and IL-4 are characterized as an additional Th subset and termed Th9 to distinguish from classical Th2 subset [28]. Th9 and IL-9 are pro-inflammatory, and appear to function in a broad spectrum of autoimmune diseases and allergic inflammation [29]. On the other hand, IL-9 may limit the capacity of antigen presenting cells (APCs) which inducing a Th1-type immune response. These observations indicate that IL-9 is a pleiotropic cytokine and the exact role of Th9 and IL-9 in AML immunity is unknown.

Here, we investigate the frequencies of these Th cells, the level of Th cells-related cytokines and mRNA expressions of key transcript factors in AML BM microenvironment, and evaluate their clinical relevance.

## Materials and Methods

### 2.1 Patients and controls

Forty-eight newly-diagnosed (ND) AML patients (23 females and 25 males; age range, 21–83 years; median age, 45 years) and 34 complete remission (CR) AML patients (15 females and 19 males; age range, 19–71 years; median age, 40 years) were enrolled in this study. AML patients were diagnosed according to French-American-British (FAB) classification system [30]. CR was defined based on International Working Group Criteria [31]. Nineteen relapsed-refractory AML patients (9 females and 10 males; age range, 18–63 years; median age, 41 years) were also enrolled in this study. These patients failed to achieve CR after two courses of standard induction chemotherapy or relapsed in 6 months after the first CR. Because BM aspiration is a quite invasive procedure, individuals with slight iron deficiency anemia, having no immunological changes, were used as controls. The control group consisted of 15 individuals (10 females and 5 males; age range, 18–67 years; median age, 39 years). Enrollment occurred between February 2012 and November 2012 in Qilu Hospital, Shandong University (Jinan, China). Characteristics of the patients at the time of sampling were provided in the Table 1. This study was approved by the Medical Ethical Committee of Qilu Hospital, Shandong University. The written informed consent was obtained from all patients before enrollment in the study in accordance with the Declaration of Helsinki.

**Table 1:** The characteristics of subjects.

	ND AML patients (n = 48)	CR AML patients(n = 34)	Relapsed-refractory AML patients(n = 19)	Controls (n = 15)
Age(years)	21–83	19–71	18–63	12–67
Gender(male/ female)	25/23	19/15	10/9	5/10
WBC(*10^9^/L)	Median: 11.45,std deviation:41.98	Median: 4.43,std deviation:2.05	Median: 5.72,std deviation: 51.8	Median: 5.5,std deviation:4.133
BM leukemic blast (%)	72.5051±41.012	1.1739±1.1541	57.2667±30.129	
FAB subtype				
M1	2	0	0	
M2	7	2	1	
M3	9	12	1	
M4	9	9	5	
M5	21	11	12	

ND = new-diagnosed; CR = complete remission; WBC = white blood cell; FAB = French-American-British; BM = bone marrow

### 2.2 Treatment regimen

Newly-diagnosed patients with non-M3 AML subtypes underwent standard induction chemotherapy with one of the anthracyclines (doxorubicin or idarubicin) for 3 days and cytarabine for 7 days. The newly-diagnosed patients with acute promyelocytic leukemia (APL, subtype M3) received all-trans retinoic acid with or without concurrent induction chemotherapy. After the patients achieved CR, they underwent consolidation chemotherapy with a conventional dose of cytarabine and one anthracycline or with a high dose of cytarabine.

### 2.3 Flow cytometric analysis of Treg cells

BM samples from all participants were collected into ethylenediamine tetraacetic acid (EDTA)-containing tubes. BM mononuclear cells (BMMCs) were isolated by Ficoll-Hypaque (Amersham Bioscience, Freiburg, Germany) gradient centrifugation and analyzed by four-color flow cytometric analysis. Briefly, BMMCs cells (1×10^6^) were surface-stained with Alexa Fluor 488-conjugated anti-CD4 (Biolegend) and Alexa Fluor 647-conjugated anti-CD25 (Biolegend) mouse monoclonal antibodies followed by incubating at room temperature in the dark for 20 minutes. Subsequently, the cells were fixed, permeablized and incubated with phycoerythrin (PE)-labelled anti-human Foxp3 (Biolegend) monoclonal antibody for 1 h in the dark. The percentages of CD4^+^CD25^+^Foxp3^+^ Tregs were determined for all participants.

### 2.4 Flow cytometric analysis of other Th subsets (Th1, Th2, Th17, Th22 and Th9)

Intracellular cytokines were studied by flow cytometry to reflex the percentages of corresponding cytokine-producing cells. Briefly, heparinized BM (400ul) with an equal volume of roswell park memorial institute (RPMI)-1640 medium was incubated for 4h at 37°C in 5% CO2 in the presence of 2.5 ng/ml of phorbol myristate acetate (PMA), 1 mg/ml of ionomycin, and 1.7 mg/ml of monesin (all from Alexis Biochemicals, San Diego, CA, USA). PMA and ionomycin are pharmacologic T cell-activating agents that mimic signals generated by the T cell receptor (TCR) complex and have the advantage of stimulating T cells of any antigen specificity. Monensin is used to block the intracellular transport mechanisms, thereby leading to an accumulation of cytokines in the cells. After incubation, 100 ul of bone marrow was taken into three flow tubes respectively, and stained with Alexa Fluor 647-conjugated anti-CD4 monoclonal antibody at room temperature in the dark for 20 minutes. After fixation and permeabilization, the cells were next stained with Fluorescein Isothiocyanate (FITC)-labelled anti-IFN-γ monoclonal antibody, PE-conjugated anti-IL-22 monoclonal antibody, and Peridinin Chlorophyll (PerCP)/Cy5.5-conjugated anti-IL-17A monoclonal antibody in the first tube, with PE-conjugated anti-IL-9 monoclonal antibody in the second tube, with PE-conjugated anti-IL-4 and FITC-conjugated anti-IFN-γ monoclonal antibodies in the third tube. All antibodies were obtained from Biolegend. Isotype controls were utilized to enable correct compensation and to confirm antibody specificity. Stained cells were analyzed by flow cytometric analysis using a FACS caliber cytometer equipped with CellQuest software (BD science Pharmingen, San Jose, CA, USA). Th22, Th17, Th1, Th2, and Th9 cells were defined as CD4^+^IFN-γ^-^IL-17^-^IL-22^+^, CD4^+^IL-17^+^, CD4^+^IFN-γ^+^, CD4^+^IL-4^+^ and CD4^+^ IL-9^+^ T cells, respectively.

### 2.5 Quantitative real-time PCR analysis of transcription factors (*RORC*, *T-bet*, *GATA-3*, *AHR* and *Foxp3)*

Briefly, total RNA was extracted with TRIzol reagents according to the manufacturer’s protocol (Invitrogen, Carlsbad, CA, USA). RNA was converted into complementary DNA (cDNA) using primerScript RT Reagent Kit (TaKaRa, Shiga, Japan). Reverse transcription reaction was done at 37°C 15min, followed by 85°C for 5s. Real-time quantitative PCR was performed in Roche Applied Science LightCycler 480II Real-time PCR systems (Roche Applied Science) in accordance to the manufacturer’s instructions. The real-time PCR system contained, in a final volume of 10ul, 3.2ul of DEPC-H_2_O, 5ul of SYBR Green Real-time PCR Master Mix, 1ul of cDNA, and 0.8ul of the forward and reverse primers. The primers of *T-bet*, *GATA-3*, *RORC*, *Foxp3*, *AHR* and the endogenous control *β-actin* were shown in Table 2. The PCR products were analyzed by melt curve analysis and agarose gel electrophoresis to determine product size and to confirm that no by-products were formed. The relative concentrations of the PCR products derived from the target gene were calculated to the number of *β-actin* transcripts used as an internal control. All experiments were conducted in triplicate.

**Table 2:** Primer sequences used for real-time PCR.

Gene	Gene bank Accession no.	Sequence
*AHR*	XM 004045136	F: 5’-CAAATCCTTCCAAGCGGCATA-3’
		R: 5’-CGCTGAGCCTAAGAACTGAAAG-3’
*T-bet*	XM 004041457	F: 5’-TTGAGGTGAACGACGGAGAG-3’
		R: 5’-CCAAGGAATTGACAGTTGGGT-3’
*GATA-3*	XM 004049045	F: 5’-CGTCCTGTGCGAACTGTCA-3’
		R: 5’-GTCCCCATTGGCATTCCTCC-3’
*RORC*	XM 004026661	F: 5’-CAATGGAAGTGGTGCTGGTTAG-3’
		R: 5’-GGGAGTGGGAGAAGTCAAAGAT-3’
*Foxp3*	XM 004064143	F: 5’-GGAAAGGAGGATGGACGAACA-3’
		R: 5’-GGAAACCTCACTTCTTGGTCCC-3’
*β-actin*	XM 003845923	F: 5’-CCTTCCTGGGCATGGAGTCCTG-3’
		R: 5’-GGAGCAATGATCTTGATCTTC-3’

### 2.6 ELISA for Th related cytokines (IL-22, IL-17, IL-6, IL-4, TGF-β, IL-10, IL-9 and IFN-γ)

BM was collected into heparin-anticoagulant vacutainer tubes. Plasma was obtained from all subjects by centrifugation and stored at -80°C for determination of cytokines. Plasma levels of IL-22, IL-17, IL-6, IL-4, TGF-β, IL-10, IL-9 and IFN-γ were determined with a quantitative sandwich Enzyme-linked Immunosorbent Assay (ELISA) in accordance with the manufacturer’s recommendations (lower detection limit 9 pg/ml; all ELISA kits from eBioscience).

### 2.7 Statistical analysis

Results were expressed as median ± SD or median (range). Statistical significance between two groups was determine by TTest if the data is normally distributed, Wilcoxon was used when the data is not normally distributed, the paired t-test was used when observing changes of the nine samples after the chemotherapy treatment. The pearson or spearman correlation test was used for correlation analysis depending on data distribution. P value<0.05 was considered statistically significant.

## Results

### 3.1 Decreased Th22 cells and plasma IL-22 levels in BM of AML patients

**Fig 1:**
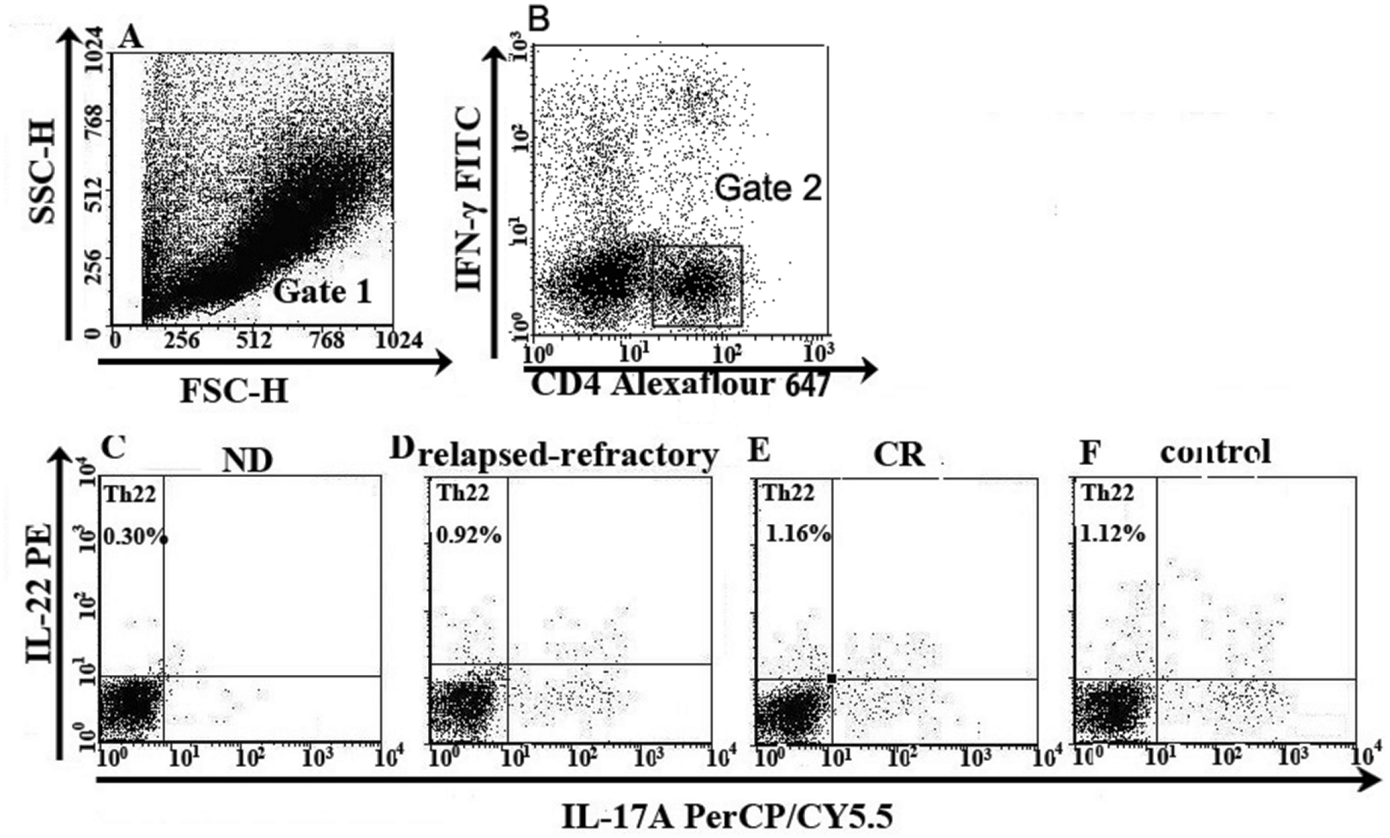
The percentage of Th22 cells in representative patients with ND, CR, relapsed-refractory AML patients or in controls. Heparinized bone marrow from all subjects was stimulated with phorbol myristate acetate, ionomycin, and monensin for 4 h or BM mononuclear cells were isolated by Ficoll-Hypaque gradient centrifugation and then stained with labeled antibodies, as described in the “Materials and methods” section. A. Lymphocytes were gated by flow cytometry. B. The percentage of CD4^+^IFN-γ^-^ T cells in AML patients and controls. C, D, E, F. The percentage of Th22 (CD4^+^IFN-γ^-^IL-17^-^IL-22^+^) cells in AML patients and controls.

**Fig 2:**
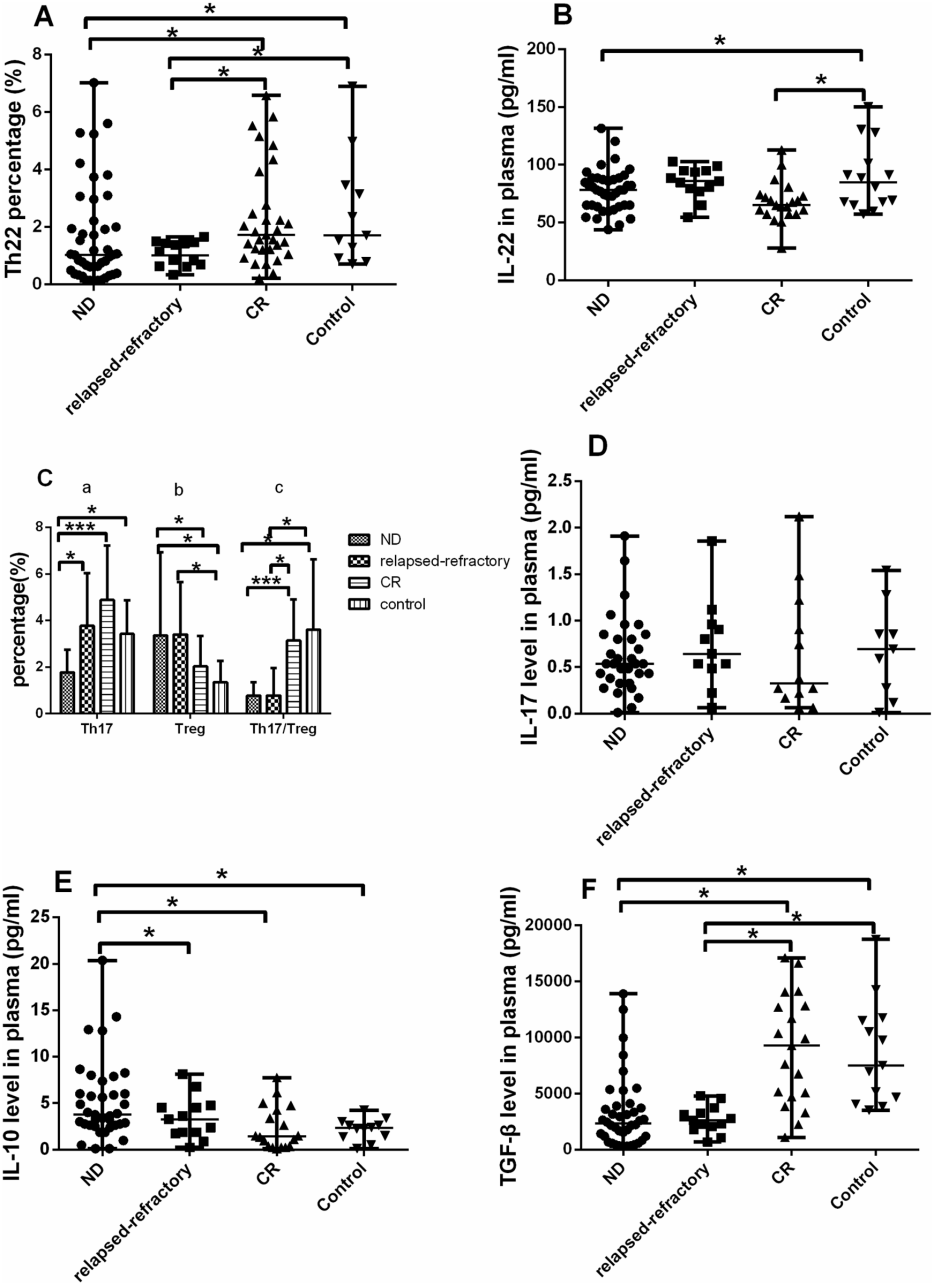
Results of Th22, Th17 and Treg cells percentages and relative cytokines. For the data is normally distributed (Th17, Treg, Th17/Treg), we use t-test to check the statistical significance. For the data is not normally distributed, we use Wilcoxon test to analysis the statistical significance. A significant decrease of Th22 percentage (A) and plasma IL-22 level (B) was observed in ND or relapsed-refractory patients (***P<0.0001, *P<0.05). C. (a). The level of Th17 cells was significantly lower in ND patients than in relapsed-refractory, CR AML patients or controls (*P<0.05; ***P<0.0001). (b, c). A significantly higher Treg percentage but lower Th17/Treg ratio was observed in ND or relapsed-refractory patients than in controls or CR patients (*P<0.05; ***P<0.0001). D. No statistical difference of IL-17 level was found in the four groups, but had an increase trend from ND stage to CR stage. E, F. A significantly higher IL-10 but a lower TGF-β were observed in ND AML patients than in controls or CR patients (*P<0.05; ***P<0.0001).

Th22 was defined as CD4^+^IFN-γ^-^IL-17^-^IL-22^+^ T cells to exclude Th1 or Th17 cells (Fig 1). Fig 1 is the representative Th22 figure. Th22 frequency in ND (median 1.03%; range, 0.14–7.02%) or relapsed-refractory AML patients (median, 1.02%; range, 0.33–1.66%) was significantly lower than that in controls (median, 2.03%; range, 0.70–19.08%, *P = 0.0263; *P = 0.0109; respectively). For the different AML stages, Th22 frequency in ND or relapsed-refractory AML patients was significantly lower than that in CR AML (median, 1.56%; range, 0.21–6.58%; *P = 0.0118; *P = 0.0067; respectively). No statistical decrease was shown in CR patients compared with controls (*P*>0.05) (Fig 2A). We also observed a significant decrease of BM plasma IL-22 level in ND patients (median, 77.63 pg/ml; range 71.73–83.53 pg/ml; *P = 0.0453) and CR patients (median, 68.28 pg/ml; range, 59.83–76.73 pg/ml; *P = 0.0340) compared with the level in controls (median, 100.78 pg/ml; range, 63.08–138.48 pg/ml). No significant difference of BM IL-22 level between the three AML groups (*P* >0.05) was observed (Fig 2B). No correlation was found between Th22 and IL-22 in BM of AML patients or controls. Meanwhile, BM Th1 and Th17 cells failed to show statistical correlations with IL-22 in our present research.

### 3.2 Decreased Th17 cells, elevated Treg cells and imbalanced Th17/Treg in BM of AML patients

**Fig 3:**
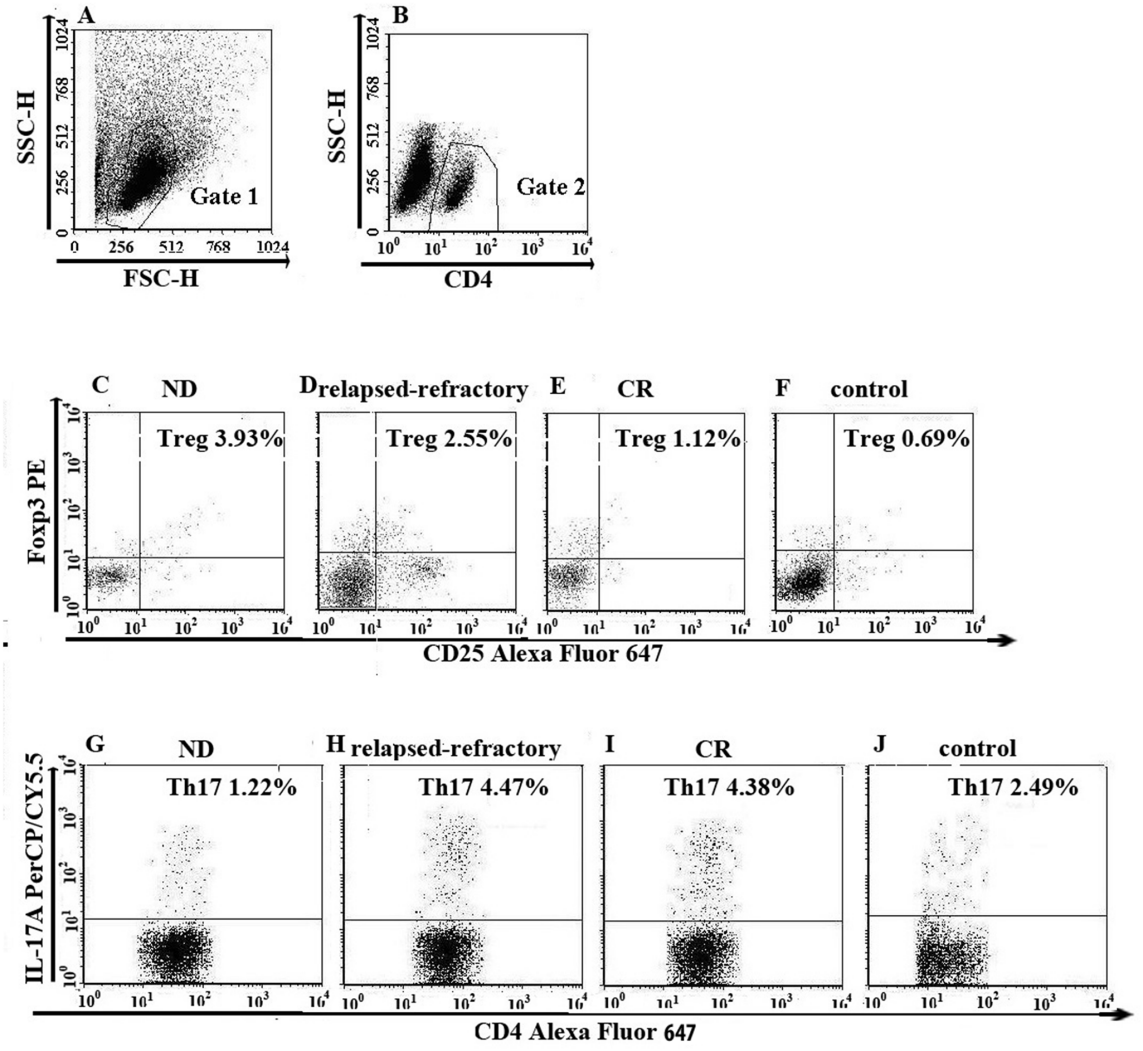
The percentage of Treg (CD4^+^CD25^+^ Foxp3^+^), Th17 (CD4^+^IL-17^+^) cells in representative patients with ND, CR, relapsed-refractory AML patients or in controls. A. Lymphocytes were gated by flow cytometry. B. The percentage of CD4^+^ lymphocytes. C, D, E, F. The percentage of Treg (CD4^+^CD25^+^ Foxp3^+^) cells in AML patients and controls. H, I, J, K. The percentage of Th17 (CD4^+^IL-17^+^) cells in AML patients and controls.

Fig 3 is the representative Th17 and Treg figure. In different stages of AML, Th17 frequency (Fig 3) was markedly decreased in ND patients (1.77 ± 0.97%) compared with the level in CR (4.88 ± 2.33%; ***P <0.0001), relapsed-refractory AML patients (3.78 ± 2.24%; *P = 0.0022) or controls (3.42 ± 1.45%; *P = 0.0001) (Fig 2C). However, no significant difference regarding BM plasma IL-17 level was found between ND AML patients (median, 0.77pg/ml; range, 0.52–1.02pg/ml) and CR (median, 0.83pg/ml; range, 0.30–1.36pg/ml) or between ND AML patients and controls (median, 0.92pg/ml; range, 0.30–1.36pg/ml) (Fig 2D). No correlation was identified between Th17 and plasma IL-17 level in all groups.

We observed the opposite performance of Treg cells in AML patients (Fig 3). Treg frequency was significantly increased in ND (3.71 ± 3.58%) and relapsed-refractory patients (3.40 ± 2.25%) compared with in controls (1.35 ± 0.91%; *P = 0.0035; *P = 0.0046; respectively). For the different AML stages, Treg frequency was significantly increased in ND and relapsed-refractory patients compared with in CR patients (1.89 ± 1.30%; *P = 0.0495; P = 0.0704; respectively) However, there is no statistical significance between CR patients and controls (P = 0.0911) (Fig 2C). Consistent with the flow cytometric results, we also found that BM plasma IL-10 level was significantly higher in ND patients (median, 6.41 pg/ml; range, 0.30–1.36pg/ml) than that in controls (median, 2.19 pg/ml; range, 1.44–2.93 pg/ml; *P = 0.0023) or CR (median, 2.13 pg/ml; range, 1.17–3.50 pg/ml; *P = 0.003) or relapsed-refractory patients (median, 3.41 pg/ml; range, 1.89–4.93 pg/ml; *P = 0.0253) (Fig 2E). There was no statistical difference between relapsed-refractory and CR patients (P = 0.1555) or controls (P = 0.1310). No correlation was identified between Treg and plasma IL-10 level in all groups.

We also investigated BM plasma TGF-β in these groups. TGF-β level was markedly decreased in ND (median, 3207.9 pg/ml; range 289.79–13883.65 pg/ml) and relapsed-refractory AML patients (median, 2691.4 pg/ml; range, 1953.2–3429.6 pg/ml) compared with the level in controls (median, 9886 pg/ml; range, 6146.2–13626 pg/ml; ***P <0.0001; *P = 0.0011; respectively). What’s more, for the different AML stages, TGF-β level was markedly decreased in ND and relapsed-refractory AML patients compared with the level in CR patients (median, 10443pg/ml; range, 6450.6–14436 pg/ml; ***P <0.0001; *P = 0.0007; respectively) (Fig 2F).

**Fig 4:**
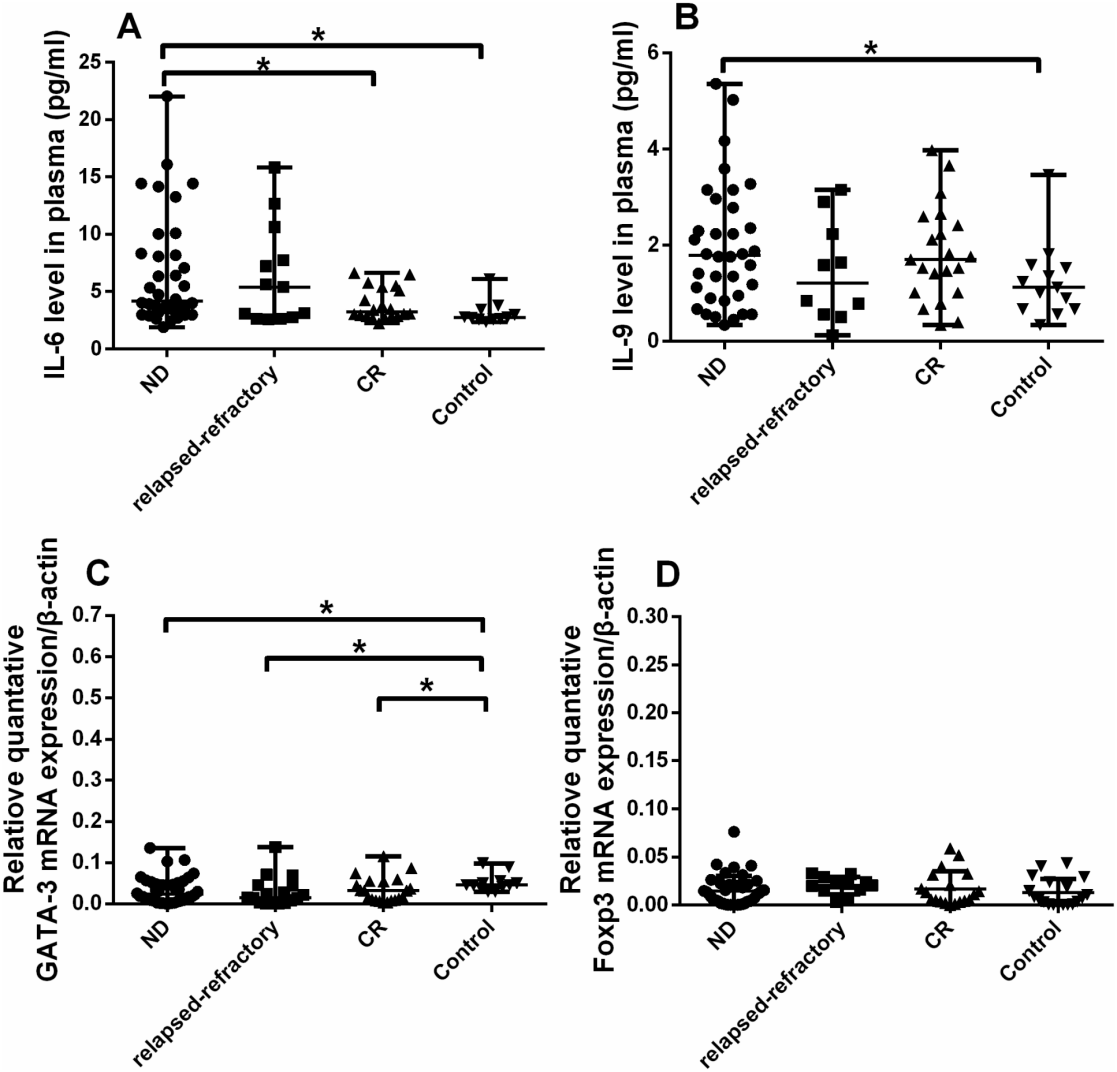
Relative cytokines (IL-6, IL-9) levels and quantitative RT-PCR results. The data is not normally distributed, so we use Wilcoxon test to analysis the statistical significance. A, B. A significantly higher IL-6 and IL-9 levels were observed in ND AML patients than in controls or CR patients (*P<0.05). C. *GATA-3* expression were decreased in ND, relapsed-refractory and CR patients compared with controls (*P<0.05). D. *Foxp3* expression was elevated in ND or relapsed-refractory patients compared to controls though no statistical significance was found.

For BM plasma IL-6 level, there was a significant increase in ND patients (median, 5.03 pg/ml; range, 1.92–175.66 pg/ml) compared to in controls (median, 2.77 pg/ml; range, 2.57–31.81 pg/ml; *P = 0.0073) or CR patients (median, 3.41 pg/ml; range, 2.63–6.64 pg/ml; *P = 0.0419). Also, we observed a marginal increase of IL-6 level in relapsed-refractory patients (median, 4.25pg/ml; range, 2.60–15.83 pg/ml) compared with controls (*P = 0.0571) (Fig 4A).

The distinct ratio imbalance of Th17/Treg was found in AML patients. Th17/Treg ratio was significantly decreased in ND (0.78 ± 0.58) or relapsed-refractory patients (0.78 ± 1.78) compared with the ratio in controls (3.61 ± 3.01; *P = 0.0226, *P = 0.0249). For the different AML stages, Th17/Treg ratio was significantly decreased in ND and relapsed-refractory patients compared with the ratio in CR (3.14 ± 1.78; ***P<0.0001, *P = 0.0011). There is no statistical difference between controls and CR AML patients or between relapsed-refractory and ND AML patients (P>0.05) (Fig 2C).

### 3.3 Decreased Th1 cells and Th1/Th2 ratio in BM of AML patients

**Fig 5:**
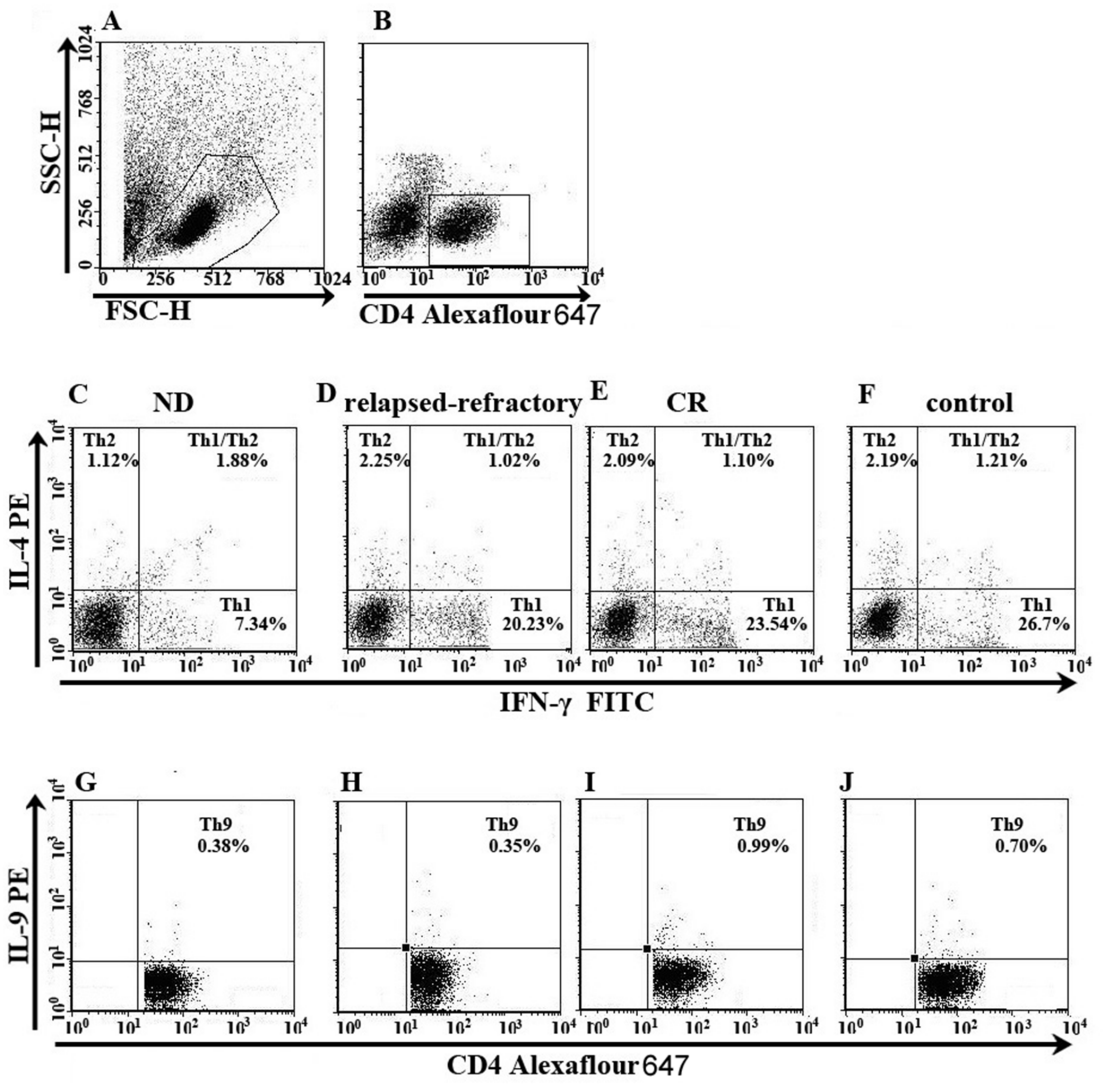
The percentage of Th1 (CD4^+^IFN-γ^+^), Th2 (CD4^+^IL-4^+^) and Th9 (CD4^+^IL-9^+^) cells in representative patients with ND, CR, relapsed-refractory AML patients or in controls. A. Lymphocytes were gated by flow cytometry. B. The percentage of CD4^+^ lymphocytes. D, E, F, G. The percentage of Th1 (CD4^+^IFN-γ^+^) and Th2 (CD4^+^IL-4^+^) cells in AML patients and controls. H, I, J, K. The percentage of Th9 (CD4^+^IL-9^+^) cells in AML patients and controls.

**Fig 6:**
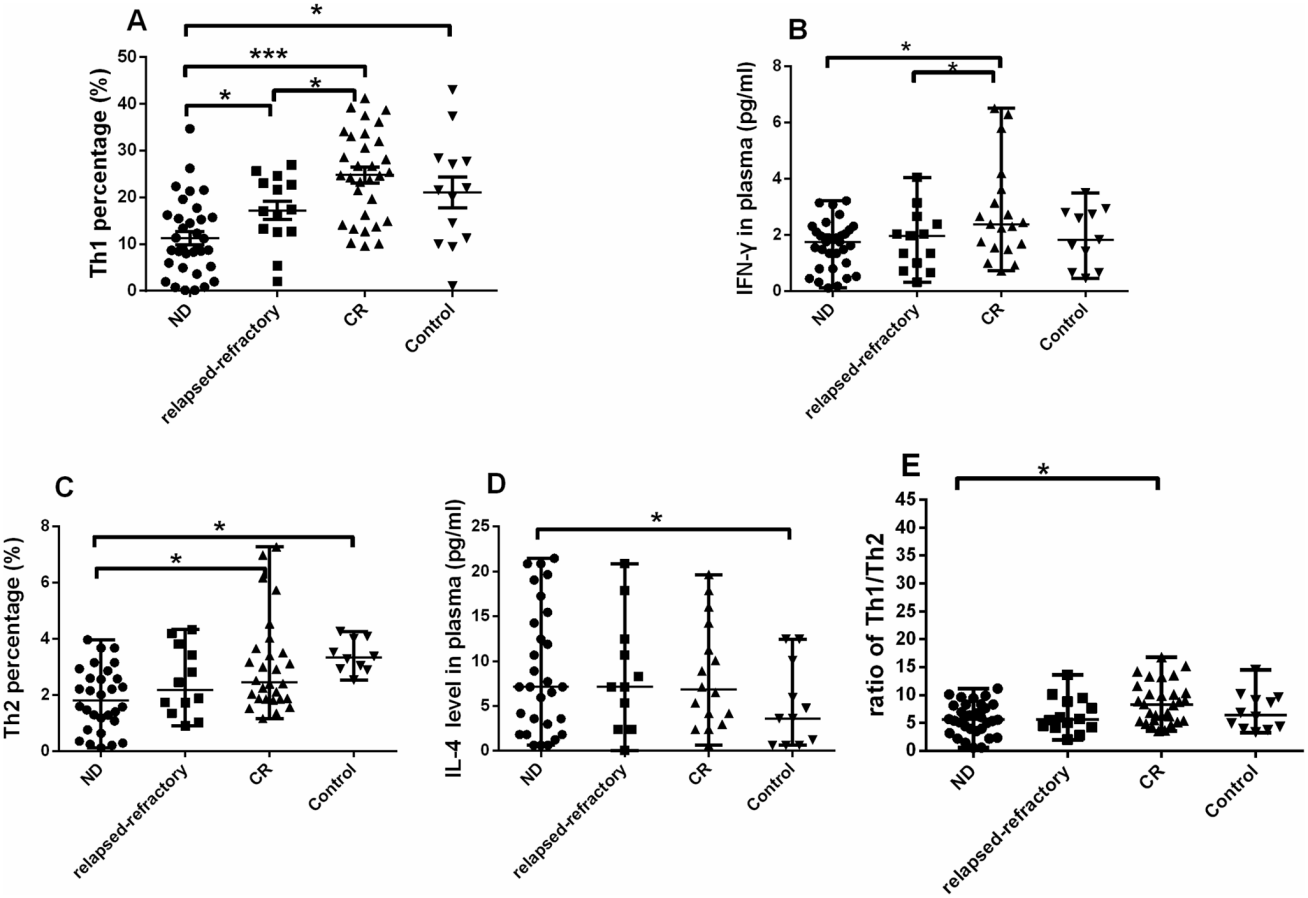
Results of Th1 and Th2 subsets. Considering the data is not normally distributed, we use Wilcoxon test to analysis the statistical significance. A, B. A significantly lower Th1 percentage and IFN-γ level was observed in ND AML patients than in controls or relapsed-refractory or CR patients (*P<0.05; ***P<0.0001). C, D. A significantly lower Th2 percentage and IL-4 level was observed in ND AML patients than in controls or CR patients (*P<0.05). E. The ratio of Th1/Th2 was significantly increased in CR stage compared with in ND AML patients.

Fig 5 is the representative Th1 and Th2 figure. Compared with controls (21.03 ± 11.83%), Th1 percentage was markedly decreased in ND patients (11.27 ± 8.11%; *P = 0.0023) (Fig 5). In different stages of AML, Th1 in ND patients was lower than that in CR (24.81 ± 9.49%; ***P<0.0001) and relapsed-refractory patients (17.23 ± 7.52%; *P = 0.0224). Also, Th1 cells frequency was significantly decreased in relapsed-refractory AML patients compared with the level in CR patients (*P = 0.0117) (Fig 6A). Consistent with flow cytometric results, we observed a significant decrease of plasma IFN-γ level in ND (median, 2.13 pg/ml; range, 1.48–2.73 pg/ml) and relapsed-refractory patients (median, 1.96 pg/ml; range, 0.31–4.05 pg/ml) compared with the level in CR (median, 2.41 pg/ml; range, 0.72–6.50 pg/ml; *P = 0.04; *P = 0.0326; respectively) (Fig 6B). No statistical difference of IFN-γ level between ND or relapsed-refractory AML patients and control group was found. No correlation was identified between Th1 and plasma IFN-γ.

For Th2 cells (Fig 5), significant decrease was found in ND (median, 2.15%; range, 0.11–10.26%) compared with CR patients (median, 2.46%; range, 0.31–11.61%; *P = 0.0425) or controls (median, 3.04%; range, 0.12–4.25%; *P = 0.0413) (Fig 6C). However, we observed a significant increase of BM plasma IL-4 level in ND patients (median, 10.83 pg/ml; range 7.11–14.55 pg/ml) compared with the level in controls (median, 5.10 pg/ml; range, 1.99–8.20 pg/ml; *P = 0.0384). No significant difference of IL-4 level between ND and CR (median, 9.97 pg/ml; range 5.47–14.46 pg/ml; P >0.05) or between ND and relapsed-refractory patients (median, 8.61 pg/ml; range 4.25–12.96 pg/ml; P >0.05) was observed (Fig 6D).

Finally, we observed imbalanced Th1/Th2 ratio in different stages of AML. Th1/Th2 ratio was markedly decreased in ND patients (5.73 ± 2.88) compared to CR patients (8.55 ± 3.69; *P = 0.0013). Also, the ratio had a decreased trend in relapsed-refractory patients (6.39 ± 3.19) compared with CR patients, even though having reached no statistical significance (P = 0.067) (Fig 6E).

### 3.4 Th9 cells and IL-9 levels in AML patients

The representative Th9 was shown in Fig 5. For Th9 cells, no statistical difference was found between these groups (P>0.05). However, we observed a statistical increase of plasma IL-9 level in ND patients compared to the level in controls (1.98 ± 1.2779 pg/ml vs. 1.25 ± 0.80 pg/ml, *P = 0.0233) (Fig 4B). Also, the IL-9 level in CR patients (1.82 ± 1.0 pg/ml) showed the elevated trend compared with in controls, even though having reached no statistical significance (P = 0.0933).

### 3.5 mRNA expressions of Th cells-related transcription factors (*AHR*, *RORC*, *Foxp3*, *T-bet* and *GATA-3)* in AML patients

*AHR*, key transcription factor directing Th22 differentiation, was determined by real-time PCR. Our result demonstrated that *AHR* was lower in BMMCs of ND (median, 0.09; range 0.05–0.14) and CR patients (median, 0.15; range, 0.11–0.22) than in controls (median, 0.15; range, 0.04–0.26), consistent with the flow cytometry and ELISA data. However, they had not reached statistical significance (P = 0.0512; P = 0.2871; respectively).

In view of decreased frequencies of Th17 in ND patients, we also detected *RORC*, the key transcription factor directing Th17 lineage commitment. *RORC* was statistically deceased in ND (median, 0.07; range 0.03–0.11), CR (median, 0.02; range, 0.0001–0.39) and relapsed-refractory AML patients (median, 0.0014; range, 0.00003–0.1871) compared with in controls (median, 0.11; range, 0.007–0.21; *P = 0.0377; P = 0.0553; *P = 0.0328; respectively).

For *T-bet*, there was no statistical difference between the four groups. *GATA-3*, key transcription factor of Th2, was significantly deceased in ND (median, 0.02; range, 0.01–0.14; *P = 0.027), CR (median, 0.03; range, 0.003–0.12; *P = 0.048) and relapsed-refractory patients (median, 0.015; range, 0.0003–0.14; *P = 0.006) compared with in controls (median, 0.05; range, 0.03–0.10) (Fig 4C).

Although there is no statistical difference between these four groups, *Foxp3* expression was elevated in ND (median, 0.011; range, 0.00004–0.04), relapsed-refractory (median, 0.02; range, 0.003–0.03) and CR patients (median, 0.01; range, 0.00004–0.06) compared with in controls (median, 0.0075; range, 0.00004–0.04), consistent with the flow cytometric and plasma IL-10 data (Fig 4D).

### 3.6 Clinical relevance of BM Th subsets in AML patients

**Fig 7:**
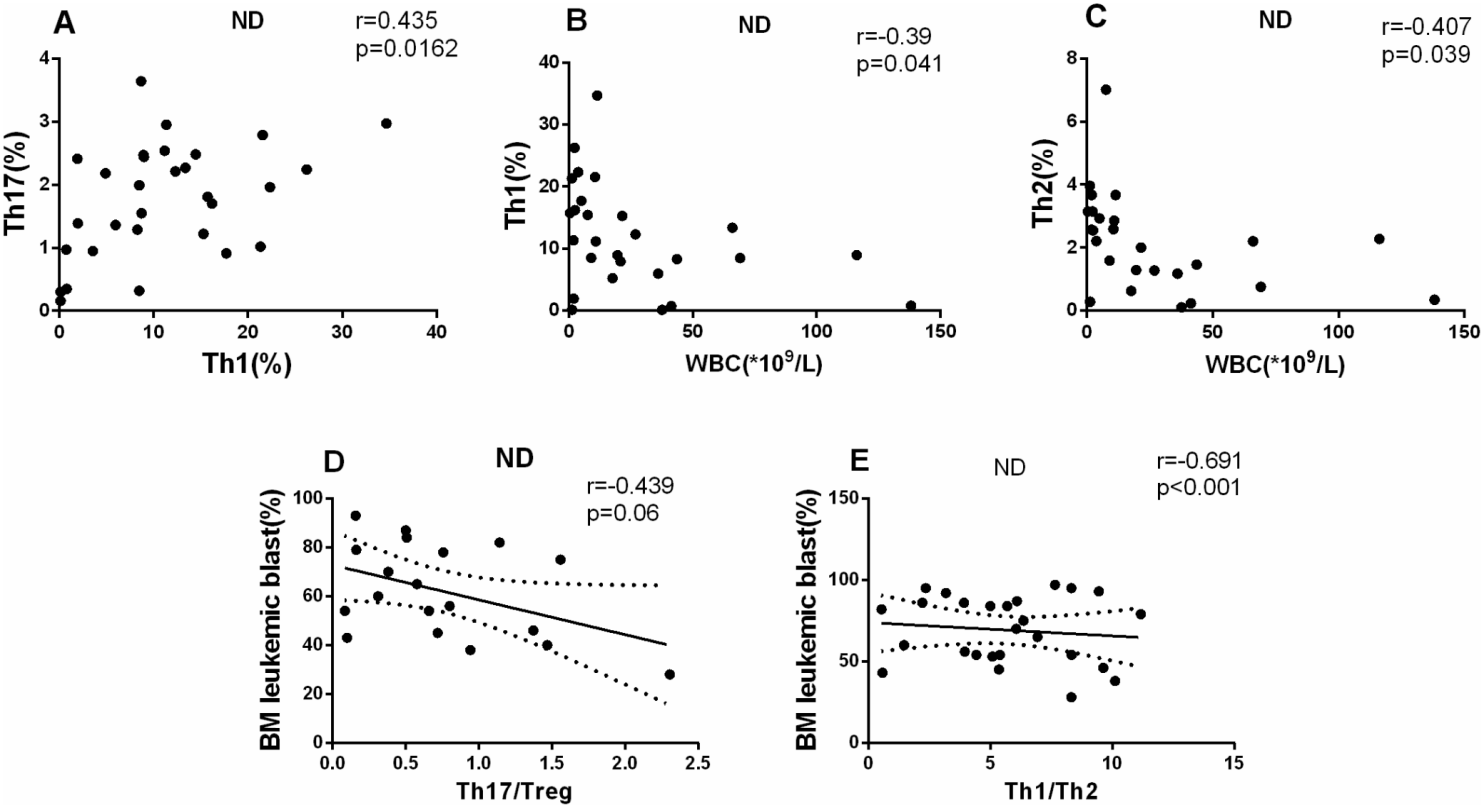
Results of correlations among Th subsets and clinical characteristics. The pearson or spearman correlation test was used for correlation analysis. A. A positive correlation was found between Th1 and Th17 cells (r = 0.435, *P* = 0.0162). B, C. A negative correlation was shown between Th1 or Th2 cells with peripheral WBC (r = -0.39, *P* = 0.041; r = -0.407, *P* = 0.039, respectively) in ND AML patients. D, E. A negative correlation was found between Th17/Treg and leukemic blast (r = -0.439, p = 0.06), Th1/Th2 and BM leukemic blast (r = -0.691, p<0.0001) in BM of ND patients.

In ND AML patients, a positive correlation was found between Th1 and Th17 (r = 0.435, P = 0.0162; Fig 7A). Moreover, we analyzed the association between the Th subsets and clinical characteristics in ND AML patients. We observed a significantly negative correlation between Th1 cells frequency or Th2 cells frequency and peripheral white blood cell (WBC) count ((r = -0.39, *P = 0.0441; r = -0.407, *P = 0.039, respectively) (Fig 7B and 7C). Also, we observed a significantly negative correlation between Th17/Treg ratio or Th1/Th2 ratio and BM leukemic blast (r = -0.439, P = 0.06; r = -0.691, ***P<0.0001, respectively) (Fig 7D and 7E).

### 3.7 Chemotherapy ameliorates imbalanced Th subsets and related cytokines in BM microenvironment of AML patients

**Fig 8:**
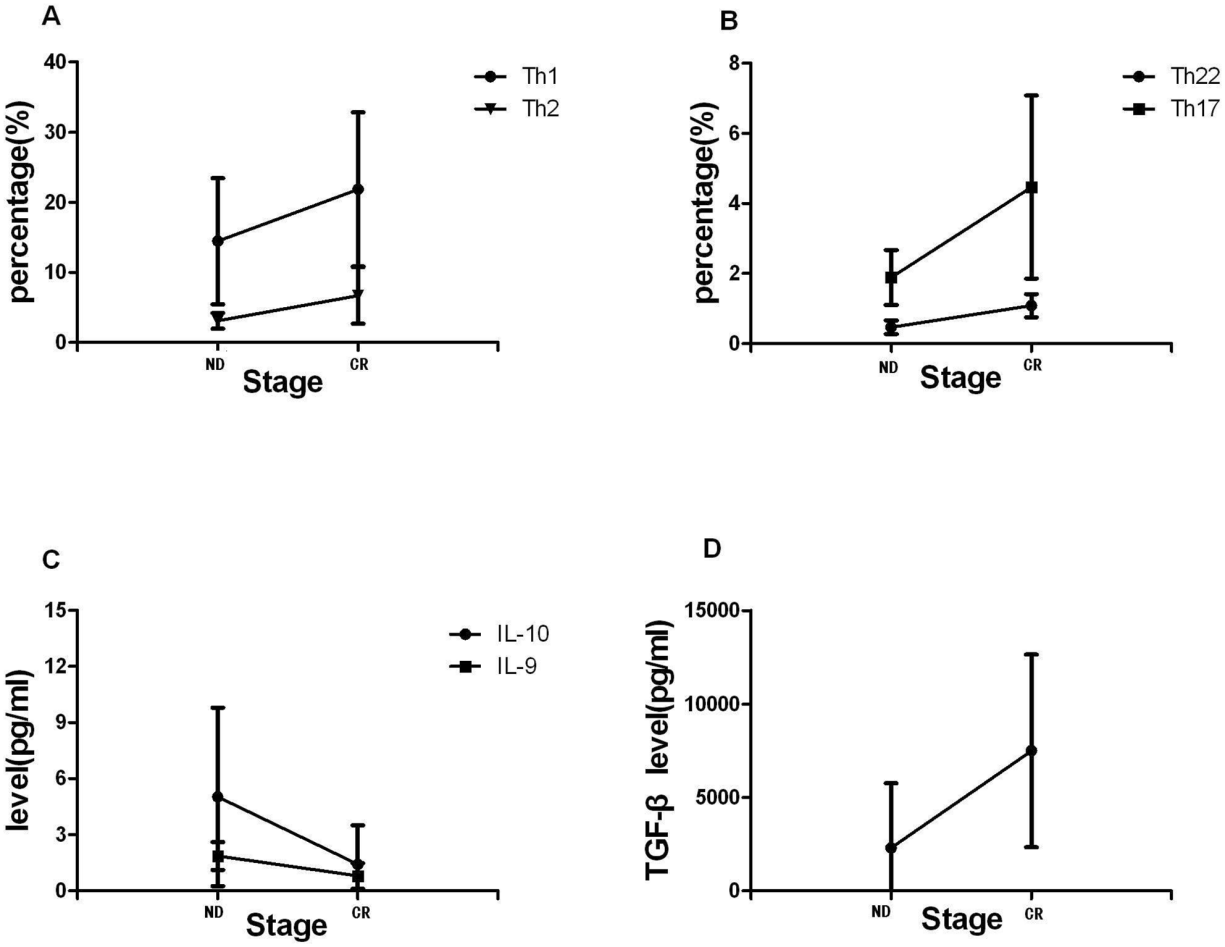
Th subsets and associated cytokines in the same AML patient cohorts. Paired t-test was used to check the changes. A,B. Th2, Th1, Th22 and Th17 cells percentages were significantly increased after chemotherapy treatment and reached the CR stage compared with the ND stage of AML. C, D. A significantly lower IL-10 and IL-9 levels, but a higher TGF-β level was observed in CR stage than in ND stage (*P<0.05).

To further understand the influence of chemotherapy on AML BM microenvironment, we observed the whole treatment process of 9 AML patients. CR was obtained after the standard induction chemotherapy. We observed a significant increase of Th1 cells (median, 24.51%; range, 5.38–38.69% vs. median, 12.87%; range, 3.6–34.67%; P = 0.0051) and Th2 cells (median, 4.34%; range, 3.46–13.2% vs. median, 3.43%; range, 1.27–5.86%; *P = 0.0313) in CR stage compared with in ND stage (Fig 8A). After chemotherapy, Th17 was also shown marginally elevated (median, 5.67%; range, 3.31–10.5% vs. median, 4.26%; range, 1.43–17.7%; P = 0.1122), as well as Th22 frequency in the patients’ CR stage compared with their ND stage (1.35 ± 0.57% vs. 0.48± 0.16%, *P = 0.0064; Fig 8B). Though the decrease of Tregs frequency did not reach statistical difference, we found a significant decrease of BM plasma IL-10 after chemotherapy (median, 1.50; range, 0.12–7.77 vs. median, 3.25; range, 1.0–20.25; *P = 0.048; Fig 8C). Plasma TGF-β was significantly elevated in CR stage (*P = 0.0298) (Fig 8D). Finally, after chemotherapy, we observed that IL-9 level was markedly decreased in CR stage (*P = 0.0447; Fig 8C).

## Discussion

In this study, not only a shift in Th1/Th2 and Th17/Treg balances, but also an aberrant Th22 subset and associated cytokines in BM microenvironment are involved in AML pathogenesis and development, and chemotherapy partly ameliorates this turmoil.

Th22 cells, a novel cell lineage, have been believed to play an important role in inflammatory, autoimmune diseases, and hematological diseases, such as MDS and ITP [24,25,32]. To study whether Th22 is compromised in the progress of AML, Th22 percentages in BM microenvironment of AML patients were determined in this study. Th22 subset frequency was markedly decreased in ND and relapsed-refractory AML patients, and recovered in CR patients. In cancers and autoimmune disorders, IL-22 expression is various [32], with IL-17 as siblings but not twins regarding their biological characteristics. Consistent with flow cytometric results, plasma IL-22 level was significantly decreased in ND or CR AML patients. Similarly, we found that *AHR* mRNA expression showed a decreased trend in AML patients compared with in controls. Moreover, through the observation of the whole treatment process of AML patients, we found that chemotherapy ameliorated the changes of Th22 subset frequency. All these results suggest that Th22 is negatively correlated to the occurrence and development of AML, and further researches are needed to elucidate the exact role of Th22 cells in AML progress.

Treg cells engage in maintaining natural self-tolerance and inhibiting immune responses by influencing the activity of other cells [11]. As known, there exists immunosuppression in the pathogenesis and progress of AML. To explore the relationship between Treg and pathophysiology of AML, we detected Tregs frequency in BM microenvironment of AML. Our results demonstrated that Treg was markedly elevated in ND and relapsed-refractory AML patients. Consistently, IL-10, secreted by Treg cells and exerting anti-inflammatory and immunosuppressive activity, was significantly increased in ND patients. Furthermore, transcript factor *Foxp3*, also presented the similar elevated mRNA expression trend. These results indicate a potential pathogenic role of Treg cells in AML. Tumor cells may recruit these Treg cells to inhibit antitumor immunity in the BM microenvironment, thus limiting the efficiency of cancer immunotherapy [33,34]. Moreover, in view of the inhibition role of IL-10, we may conclude that Tregs accumulating in the BM of AML patients mediate vigorous suppression via IL-10. After chemotherapy, the BM plasma IL-10 was markedly decreased, which indicated IL-10 can be a negative effect factor.

Th17 and their effector cytokines are being recognized as important mediators in autoimmune diseases, but their specific roles in tumor immunity, especially in AML BM microenvironment are rarely investigated. Our results demonstrated that Th17 was markedly decreased in ND patients. Consistently, plasma IL-17 was also shown a decreased trend in ND patients. *RORC*, the key transcription factor regulating Th17 differentiation, was accordingly decreased in ND and relapsed-refractory patients, which might give rise to the changes of Th17 and IL-17. Moreover, in the same cohort of AML patients it showed that decreasing Th17 partly restored after standard chemotherapy, indicating the importance of Th17 in AML pathogenesis. However, interestingly, in patients with relapse/refractory disease, the Th17 cell percentage is higher than ND patients and same as CR. The possible explanation is as follows. On the one hand, in relapsed-refractory AML patients, we observed that both Th17 cells and Th1 cells are increased compared with ND AML patients. It is probably that compared with the immune-suppression status in ND patients, the immune status of relapsed-refractory patients might increase because of the chemotherapy. On the other hand, there are only 19 relapsed-refractory patients in our study, which might affect the statistical analysis. Therefore, larger volume of patients is needed to clarify their similarity and difference.

What’s more, studies have demonstrated that Th17 cell frequency was increased in peripheral blood samples from untreated patients with AML compared to healthy volunteer along with increased IL-17 [21]. The conflicting data in peripheral blood and in BM microenvironment that showed in our study may be explained. The possible reason is that the two studies’ control groups are different. In the peripheral blood study, the control group is the healthy control. However, considering that bone marrow aspiration is a quite invasive procedure, individuals with slight iron deficiency anemia, having no immunological changes, were used as controls in the BM study. These two types of controls may have different levels of Th cells. Moreover, AML is a type of diseases that are originated from the BM. The clinical symptoms of AML will firstly be shown in BM, then after a period of time, the changes would be demonstrated in the peripheral blood. Therefore, it may be conflicting between the BM and peripheral blood studies.

The imbalance of Th17/Treg correlated with the existence and development of tumors and autoimmune diseases, so we evaluated the shift of Th17/Treg in bone marrow of AML patients. The ratio of Th17/Treg was significantly decreased in ND or relapsed-refractory AML patients and negatively related to the leukemic blast in ND AML BM. These all imply that the imbalance of Th17/Treg may play a pathological role in AML pathophysiology and can be used as a target for further researches and future therapeutic intervention.

TGF-β, controlling various cellular functions, is the main regulator for Th17 and Treg differentiation. Our results demonstrated that TGF-β was significantly decreased in ND or relapsed-refractory AML patients, and significantly elevated after chemotherapy, which implied that TGF-β may play a positive effect in AML progress.

Th1 cells, mediating cellular immunity, activate CD8^+^ CTL cells and promote reproduction through secreting IL-2 and IFN-γ. On the contrary, Th2 cells evolve to enhance humoral immunity and allergic response. The balanced Th1/Th2 plays an important role in normal immune system. And accumulating evidences indicate the imbalance ratio of Th1/Th2 is involved in many diseases, including the hematologic malignancies. So we detected the frequencies of Th1 and Th2 in AML BM microenvironment to investigate the possible role of Th1/Th2 shift. Our results demonstrated that Th1 and IFN-γ level were both markedly decreased in ND patients, which may imply a down-regulated cellular immunity in AML BM microenvironment. Different from the peripheral studies, a decreased trend of Th2 as well as *GATA-3* expression was observed in ND patients, and markedly elevated in CR stage after chemotherapy, indicating the defect of humoral immunity in AML. However, IL-4, Th2 effector cytokine, was significantly elevated in ND patients, which may be due to the fact that IL-4 was also secreted by the naïve Th cell itself upon Notch triggering [35] or by mast cells and macrophages. Moreover, TGF-β plays an inhibitory role on IL-4, and in view of decreasing level of TGF-β, it may be easy to explain the increased IL-4 level in BM plasma [36]. What’s more, we showed that Th1 and Th2 cells frequencies had a negative relevance to the peripheral WBC counts. Furthermore, the ratio of Th1/Th2 was significantly decreased in ND patients and negatively related to the leukemic blast in AML BM. What’s more, imbalanced Th1/Th2 ratio can be recovered when patients sensitive to chemotherapy, while not when failure to chemotherapy. Further studies are needed to explore the specific role of BM Th1/Th2 in AML tumorigenesis and progress.

In our study, there existed a positive correlation between Th17 and Th1 in ND patients, implying that they may be induced in a same influential manner in AML. Such functional synergism may happen in the persistent immunosuppressive status of AML patients, in which increased Tregs, dysfunctional NK cells, decreased dendritic cell to present antigen and against tumor cells, and higher immunoinhibitory molecule expressions resulting in immune evasion of the malignant clone are widely recognized features.

Accumulating evidences showed that Th9 and IL-9 have pleiotropic functions on the immune system and play different roles in different situations [37]. Our results showed no Th9 difference between AML patients and controls, while IL-9 level in ND patients was markedly increased. Besides being secreted by Th9, IL-9 was first associated with Th2, and also produced by Th22 and Th17 [35]. More importantly, IL-9 appeared to be a key molecule that affects both differentiation of Th17 cells and Tregs. IL-4 combined with TGF-β greatly enhanced IL-9 production, which may explain the inconsistent consequence about IL-9 and Th9. As studies demonstrated that IL-9 enhanced the suppressive function of Treg in vitro and the absence of IL-9 signaling weakened the suppressive activity of Treg in vivo [38], IL-9 may play a immunopathological role in AML. Our study also showed that BM plasma IL-9 was significantly decreased after chemotherapy, which further indicated the suppressive effect of IL-9 on AML immune microenvironment.

IL-6, produced by a variety of cells, affects the differentiation of various immune cells. Our previous study showed that IL-6 in peripheral blood of AML patients was higher than controls [39]. Consistently, we demonstrated that the BM plasma IL-6 level was also markedly elevated in ND AML patients, which indicated that there is an immune disorder, which may affect AML progress through effect factors. The exact role of cytokine IL-6 needs to be elucidated.

In summary, the down-regulation of Th22, Th17, Th1 and their secreted cytokines, the up-regulation of Treg and immunoinhibitory cytokines, the shift of Th17/Treg and Th1/Th2, and the aberrantly-expressed transcription factors may synergistically contribute to the suppressive immune system in AML. And standard induction chemotherapy may partly ameliorate these abnormal changes. Further studies are awaited to clarify their specific roles in the immunopathology of AML occurrence and development, finally provide the perspective for clinical treatment.
